# Nuclear receptor *nhr-48* is required for pathogenicity of the second stage (J2) of the plant parasite *Meloidogyne incognita*

**DOI:** 10.1038/srep34959

**Published:** 2016-10-20

**Authors:** Chao-Jun Lu, Bao-Yu Tian, Yi Cao, Cheng-Gang Zou, Ke-Qin Zhang

**Affiliations:** 1 State Key Laboratory for Conservation and Utilization of Bio-Resources in Yunnan, Yunnan University, Kunming, Yunnan 650091, China; 2 College of Life Science, Fujian Normal University, Fuzhou, Fujian 350108, China; 3 Key Laboratory of Molecular Genetics, Guizhou Academy of Tobacco Science, Guiyang, Guizhou 550081, China

## Abstract

Nuclear receptors (NRs) are a diverse class of transcription factors, which are involved in regulating a large number of physiological events in metazoans. However, the function of NRs is poorly understood in plant-parasitic nematodes. Here, members of the NR1J+K group of NRs in nematodes, including the free-living and plant parasites, were examined and phylogenetically analyzed. We found that the number of members of the NR1J+K group in plant-parasitic nematodes was less than that in the free-living nematodes, suggesting this reduction of NR1J+K group members in plant parasites maybe arose during the separation of the free-living and intermediately plant parasitic nematodes (*Bursaphelenchus xylophilus*). Interestingly, the DNA-binding domain (DBD) and ligand-binding domain (LBD) of NR1J+K members were separated into two gene locations in the plant parasites. Knockdown of *Meloidogyne incognita WBMinc13296,* the ortholog of *Caenorhabditis elegans nhr-48* DBD, reduced infectivity, delayed development, and decreased reproductivity. J2 of *M. incognita* subjected to silencing of *WBMinc13295,* the orthologs of *B. xylophilus nhr-48* LBD, exhibited developmental lag within the host and reduced reproductivity. This study provides new insights into the function of NRs and suggests that NRs are potential targets for developing effective strategies for biological control of plant-parasitic nematodes.

Plant-parasitic nematodes are major pests of numerous plants and represent a real threat to the sustainable development of worldwide crop production^1^. Among plant-parasitic nematodes, the sedentary endoparasitic genus *Meloidogyne* spp., also known as root-knot nematode (RKN), is the most damaging crop pathogen^2^. RKNs feed on the plant hosts and complete most of their life cycle inside the plant root tissues^3^. Beyond the traditionally soil-based strategies to control RKN (eg. chemical agents), the alternative is to produce RKN-resistant cultivars through genetic engineering on the basis of naturally host-derived resistant genes. To date, several available lines of tomato and rootstock cultivars with expression of *Mi-1* or its derived genes, which were isolated from a wild relative *Lycopersicon esculentum*^4^, have confered resistance to *Meloidogyne* spp. In addition, several cystatin (cysteine proteinase inhibitor) genes existed in numerous plant species have then introgressed into some crops^5^,^6^. These cystatin genes have been shown to confer resistance to several economically important plant parasites including root-knot nematodes^7^,^8^. On the other hand, bioengineering crops expressing dsRNAs that silence target RKN parasitism genes is another viable strategy to control RKN^9^.

RNA interference (RNAi), first elaborated well in the free-living nematode *Caenorhabditis elegans*, has developed as a powerful tool for gene silencing in various eukaryotic organisms^10^. Owing to their obligatory parasitic nature, the second-stage (J2) of *Meloidogyne* spp. is the crucial stage for RKN pathogenicity and development. Previous studies have shown that the J2 of RKNs subjected to gene silencing by RNAi display correspondingly obvious changes in phenotype^9^,^11^,^12^,^13^,^14^. Recently, some genes that are involved in nematode parasitism and development, such as integrase and splicing factor genes, tyrosine phosphatase gene, putative parasitism gene *16D10* and *8D05* etc., have been used to develop transgenic plants to reduce the infestation of RKNs^15^. When J2 of RKN feed on these transgenic hosts, the downregulation of the targeted gene appears to be transmitted to their offspring^16^. Success in the application of host-induced gene silencing strategies through *in vitro* RNAi has paved the way for developing a control strategy to target the various RKNs in economically important plants.

As a very ancient subfamily of Nuclear receptors (NRs), the NR1J+K NR group, including DAF-12, NHR-8 (both having DBD and LBD), and NHR-48 (only containing DBD), generally contains a well-conserved DNA-binding domain (DBD) and a variable ligand-binding domain (LBD). It is well known that members of NR1J+K play the crucial roles in development, reproduction, feeding and pathogenicity of free-living and animal-parasitic nematodes. For instance, DAF-12 governs the ability of *C. elegans* to alternate entry into and recovery from the dauer larvae stage in response to nutrient and environmental challenges. Under favorable conditions, dafachronic acids (DAs), generated from cholesterol through a multi-step pathway involving the *daf-9* cytochrome P450 enzyme, bind and activate DAF-12, leading to dauer recovery^17^,^18^. Under adverse conditions, DAF-12 together with its co-repressor DIN-1 promotes the entry of larvae into dauer diapause in the absence of DAs. Despite occupying different ecological niches, the life cycles of various species of nematode are generally highly conserved, including egg, four larvae or juvenile stages separated by molts, and adult. It has long been accepted that parasites evolved from free-living nematodes and dauer larvae work as a facilitator of phenotypic evolution towards parasitism^19^. The molecular mechanism controlling *C. elegans* larvae entry into diapause has been proved to be evolutionarily conserved and governs development of the stage 3 infective larvae (iL3) in animal-parasitic nematodes, including *Strongyloides stercoralis, Ancylostoma* spp., and *Necator americanus*^20^,^21^. Through activating an ortholog of the nuclear receptor DAF-12, application of DAs remarkably restrains the dauer-like pathogenic iL3 population in *S. stercoralis*, resulting in decreased infectivity^20^. As for facultative plant parasites, Wang *et al*.^22^ demonstrated that the canonical ortholog of *C. elegans daf-12* is evolutionarily conserved in *Bursaphelenchus xylophilus*, a destructive pathogen of pine. Treatment of *B. xylophilus* with DA significantly lowers the proportion of dispersal L4 formation, in turn resulting in propagative L4 and adults^23^. In addition, as a member of the NR1J+K group, *nhr-8* has been demonstrated to control dauer formation by being engaged in cholesterol balance and bile acid metabolism^24^. Loss of *nhr-8* results in deficiency in producing DA and controlling entry into the dauer stage, and also reduces fertility and shortens lifespan. *C. elegans* LBD-free *nhr-48*, expressed in the pharyngeal gland of larvae and adults and also in the spermatheca beginning in L4, is required to restrict the expression of gland-specific genes, implying that *nhr-48* play a role in feeding^25^,^26^.

Like *C. elegans*, plant parasite root-knot nematodes (*Meloidogyne* spp., RKN) have a simple life cycle including egg, four juvenile stages separated by molts, and adult. After undergoing a second molt inside the eggs, hatched, pre-parasitic J2s which are homologous to iL3 of animal parasites, penetrate host plant roots. Interestingly, J2s exhibit diapause as a nonfeeding, dormant filariform with a sealed buccal capsule and thickened body wall cuticle, which resemble the dauer larvae of *C. elegans* and iL3 of animal parasites in striking morphological features. Like the dauer larvae of *C. elegans*, J2s depend on the glyoxylate pathway^27^, and recover from diapause development after penetrating into the root and feeding. However, to date, there is no report or information concerning about the function of the members of the NR1J+K group in obligate plant parasites. Here, we focus on exploring *nhr-48*, which is the only member of the NR1J+K group identified in two common *Meloidogyne* species (*M. incognita* and *M. hapla*) to demonstrate its roles in infection, mobility, development, and fecundity of the plant parasite.

## Results

### Bioinformatics analysis of the group of NR1J+K homologues in nematodes

**Figure 1:**
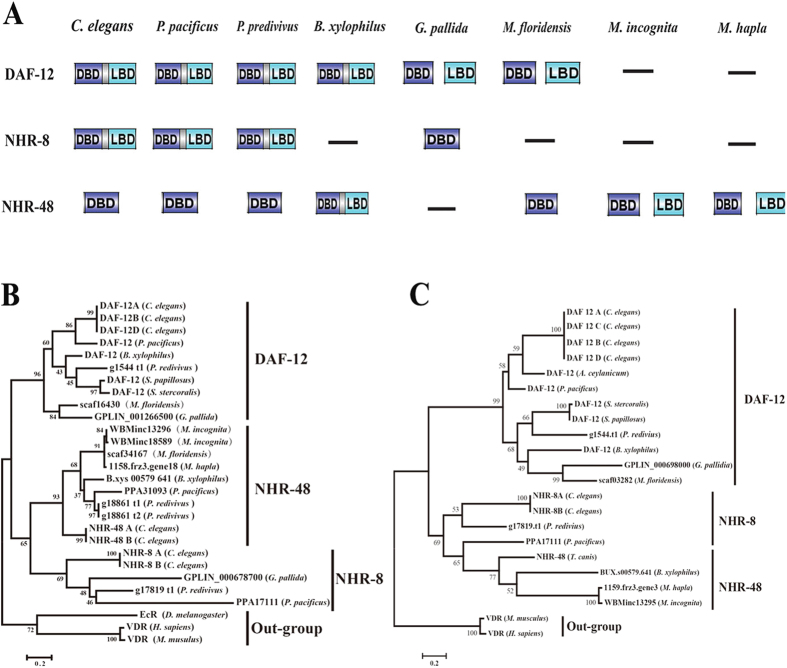
The group of 1J+K NHRs in nematodes. (**A**) The homologues of DAF-12, NHR-8 and NHR-48 in nematodes, the box labeled with DBD and LBD presented the corresponding domain, respectively. The solid line indicates the absence of corresponding homologues. (**B**,**C**) Phylogenetic relationship of 1J+K NHRs in nematodes based on Maximum-likelihood phylogeny using DBD and LBD, respectively. DAF-12 (*S. stercoralis*): 3GYT_A, DAF-12 (*S. papillosus*): ACN32203, DAF-12 (*A. ceylanicum*): 3UP0_A, NHR-48 (*T. canis: Toxocara canis*): KHN74573.

Using the corresponding DBD and LBD protein sequences of NR1J+K of *C. elegans*, we performed reciprocal BLAST searches to identify homologues in the proteomes of several nematodes including *M. incognita, M. hapla, M. floridensis, Globodera pallida, Pristionchus pacificus, B. xylophilus* and *Panagrellus redivivus*. To confirm hits, we performed reverse BLAST searches against the proteome of *C. elegans*. We identified canonical DAF-12 homologues in *P. redivivus, P. pacificus* and *B. xylophilus*, which are consistent with previously reports^22^,^28^,^29^. DAF-12 homologues in three plant parasites (*B. xylophilus, G. pallida* and *M. floridensis*) shared significant identities with *C. elegans* DAF-12 (CeDAF-12) in their corresponding DBD and LBD (Fig. 1A). Consistent with previous annotation by Cotton *et al*.^30^, two uncanonical DAF-12 homologues, whose two conserved domains (DBD and LDB) are evolutionally separated and detached into two genes, were identified in *G. pallid* and *M. floridensis*, respectively. However, we failed to explicitly identify canonical and uncanonical DAF-12 homologues in *M. incognita* and *M. hapla* with published genomes. Furthermore, we also found homologues of NHR-8 in *P. redivivus* and *P. pacificus* with high identities to *C. elegans* NHR-8 (CeNHR-8) (Fig. 1A), and homologues of NHR-8 without LBD in *B. xylophilus*. NHR-8 homologues have not been found in three species of *Meloidogyne (M. floridensis, M. incognita* and *M. hapla*) on the basis of their corresponding genes prediction to date.

As for NHR-48, we found that CeNHR-48 homologues existed in all nematodes except for *G. pallida* and contained the base-contact residues (ESCKAFFR) unique to the NR1J+K group. In addition, the CeNHR-48 homologue possessed highly conserved DBD consisting of two C4-type zinc fingers, one containing a group of four Cys residues, the other identical to that of DAF-12 and NHR-8 (Fig. S1B). Owing to the absence of the CeNHR-48 LBD, using the LBD protein sequences of CeDAF-12 and CeNHR-8 to BLAST against plant parasitic nematode proteomes, we found two orphan receptors, WBMinc13295 and 1159.frz3.gene3, both only containing family1 LBD homologous to those of CeNHR-8 and CeDAF-12 in *M. incognita* and *M. hapla*, respectively (Fig. S1C). Strikingly, consistent with the previous report by Abad^3^, the ortholog of *C. elegans nhr-48* had two copies or alleles in *M. incognita*, namely *WBMinc13296* and *WBMinc18589*, which showed high identities with each other in nucleotide and amino acid sequences (97.7% and 98.5%, respectively). Moreover, the orphan receptor *WBMinc13295* was next to *WBMinc13296*, one of LBD-free *nhr-48* orthologs, at the chromosome level where they were localized in two adjacent genetic loci in the same contig. However, *WBMinc18589*, the other *nhr-48* ortholog, was located in another contig (Fig. S1A). Whether a similar phenomenon exists in *M. hapla* with the *1159.frz3.gene3* and *1158.frz3.gene18* existing together in two adjacent genetic loci cannot be confirmed until the availability of a high quality *M. hapla* genome, though the genes are separated by an unidentified regulator of *Streptomyces* spp and localized in two adjacent contigs (Fig. S1A).

Next, to further identify members of NR1J+K, maximum likelihood (ML) phylogenetic trees of DBD and LBD domain sequences were constructed using Mega6.0^31^. ML analysis of DBD sequences revealed that NHR-48, NHR-8 and DAF-12 were clustered in different clades (Fig. 1B). Consistent with the above and previous results^30^, DBD of CeDAF-12 homologues (scaf16430) of *M. floridensis* and (GPLIN_126650) of *G. pallid* were clustered into the group of DAF-12 with a high bootstrap. Similarly, DBD of CeNHR-8 homolog (GPLIN_000678700) of *G. pallida* was clustered into the line of NHR-8 of *C. elegans* with a relatively lower bootstrap. In addition, DBD of CeNHR-48 homologues *WBMinc13296* of *M. incognita, 1158.frz3.gene18* of *M. hapla*, and scaf34167 of *M. floridensis* were strongly clustered into the group including NHR-48 of *C. elegans* with a high bootstrap.

As expected, ML-based data analysis of LBD domain sequences also revealed a phylogenetic tree similar to that of DBD (Fig. 1C). The tree displayed that LBD of CeDAF-12 homolog scaf03282 of *M. floridensis* and GPLIN_698000 of *G. pallida* were clustered into the line of CeDAF-12 with a high bootstrap and they were relatively close to DAF-12 of *B. xylophilus*. However, the two orphan receptors homologous to the LBD of CeDAF-12 and CeNHR-8, WBMinc13295 of *M. incognita* and 1159.frz3.gene3 of *M. hapla* were clustered into the line containing *B. xylophilus* BUX.s00579.641 belonging to NHR-48, implying that WBMinc13295 and 1159.frz3.gene3 are LBD orthologs of *B. xylophilus* NHR-48. MP-based data analysis of DBD and LBD resulted in tree topology comparable with the ML tree, although the number of resolved nodes was lower (data not shown). Together, the above phylogenetic analysis strongly supported the previous results of reciprocal BLAST searches. Thus, these results imply that NR1J+K in nematodes show co-evolution with the divergent evolution of ecological niches.

### Knockdown of *nhr-48* orthologs *WBMinc13296* and *WBMinc13295* in *M. incognita*

To explore the biological function of *nhr-48* orthologs in *M. incognita, WBMin13296* and *WBMin13295* were knockdown in the infectious J2 of *M. incognita*, respectively. Although the pre-parasitic J2 of *M. incognita* are essentially non-feeding, several studies have successfully demonstrated the ingestion of dsRNA or siRNA through employing resorcinol as a stimulant *in vitro*^9^,^12^,^13^. However, resorcinol at 1% concentrate incubation with J2s for a period of 4 h or more was lethal to a proportion of the worms^32^. We found that worms incubated with 1% resorcinol for 2 h and additionally incubated with buffer with FITC for 24 h exhibited a markedly higher intensity of FITC and survival rate than those incubated with 1% resorcinol for 4 h as described by Huang *et al*.^9^ (Fig. S2A–C). Thus, the modified method was employed to perform RNAi in *M. incognita*. We found that both dsRNA and siRNAs of *WBMinc13296* and *WBMinc13295* elicited specific silencing of their corresponding targets, leading to a significant reduction in transcript abundance (Fig. S3A–C).

### NHR-48 orthologs are not required for nematode attraction by host roots

**Figure 2:**
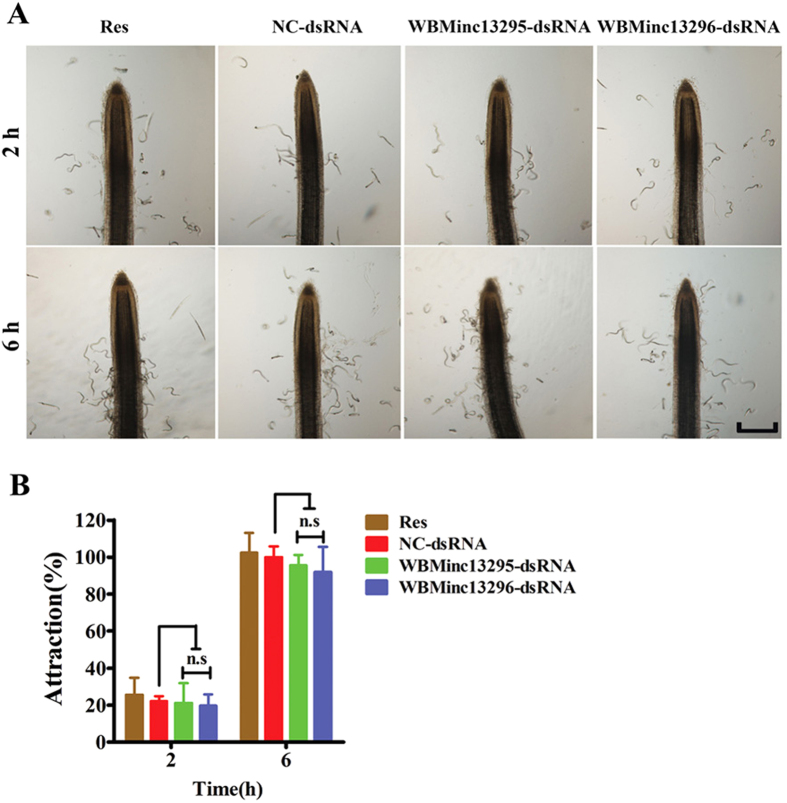
Attraction of the J2s treated with dsRNA of *WBMinc13295* and *WBMinc13296* in Pluronic gel. (**A**) photographs displayed attraction of the treated J2s response to tomato root tip at period of 2 h and 6 h (scale bar, 400 μm). (**B**) Both *WBMinc13296* and *WBMinc13295* silenced J2s did not exhibited obvious difference in response to host root tip compared to control J2s. Each bar value represents the mean ± SD of triplicate experiments (Two-way ANOVA, ns: no significance).

Prior to invasion, J2s of *Meloidogyne* spp. are required to locate the host through perceiving secretions from the host root^33^. In order to test whether *WBMinc13296* and *WBMinc13295* were involved in attraction of nematodes by the root tip, attraction assays of *M. incognita* J2s were employed with Pluronic gel medium as described previously^34^. Consistent with a previous observation^35^, *M. incognita* J2s randomly dispersed throughout the gel 2 h after the attraction assay. The number of J2 emerging in the area of 1 mm around a tomato root tip peaked at 6 h after assay initiation, followed by a decrease in number of J2 (Fig. 2A,B). However, knock-down of either *WBMinc13295* or *WBMinc13296* failed to influence the attraction of J2s to host root tips (Fig. 2A,B, Fig. S4A–D).

### *WBMinc13296*, but not *WBMinc13295*, is involved in infection of host root by J2 of *M. incognita*

**Figure 3:**
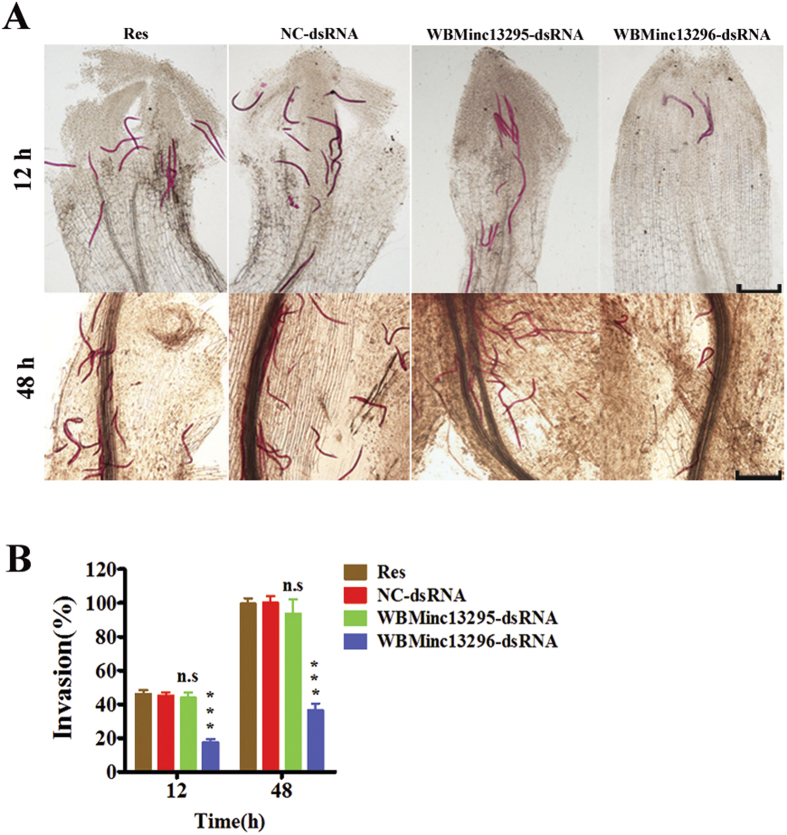
The invasion of the J2s treated with dsRNA of *WBMinc13296* and *WBMinc13295* in Pluronic gel. (**A**) photographs displayed J2s within tomato root at of period of 12 h and 48 h (scale bar, 300 μm). (**B**) *WBMinc13296* other than *WBMinc13295* silenced J2s displayed significantly reduced invasion rate of host root. Each bar value represents the mean ± SD of triplicate experiments (Two-way ANOVA, ns: no significance, ***P < 0.001).

To examine the role of *WBMinc13295* and *WBMinc13296* in the invasion of the host root, tomato roots were stained with acid fuschin to permit counting of J2s after infection. J2s treated with dsRNA of *WBMinc13296* displayed a significant reduction in infection rate in tomato roots at 12 h and 48 h of infection. In contrast, knock-down of *WBMinc13295* did not influence the infection of tomato roots with J2s (Fig. 3A,B). Intriguingly, J2s subjected to siRNA of *WBMinc13296* or *WBMinc13295* exhibited resembled results (Fig. S5). It should be noted that worms treated with *WBMinc13296* dsRNA migrated considerably faster than worms treated with NC-dsRNA (Fig. S6). Thus, it is unlikely that the decrease in infection of root by knock-down of *WBMinc13296* is due to lower migratory activity. Together these data suggest that the knock-down of *WBMinc13296* significantly reduces the infection by J2s.

### Inactivation of *WBMinc13296* or *WBMinc13295* suppresses pathogenicity of J2

**Figure 4:**
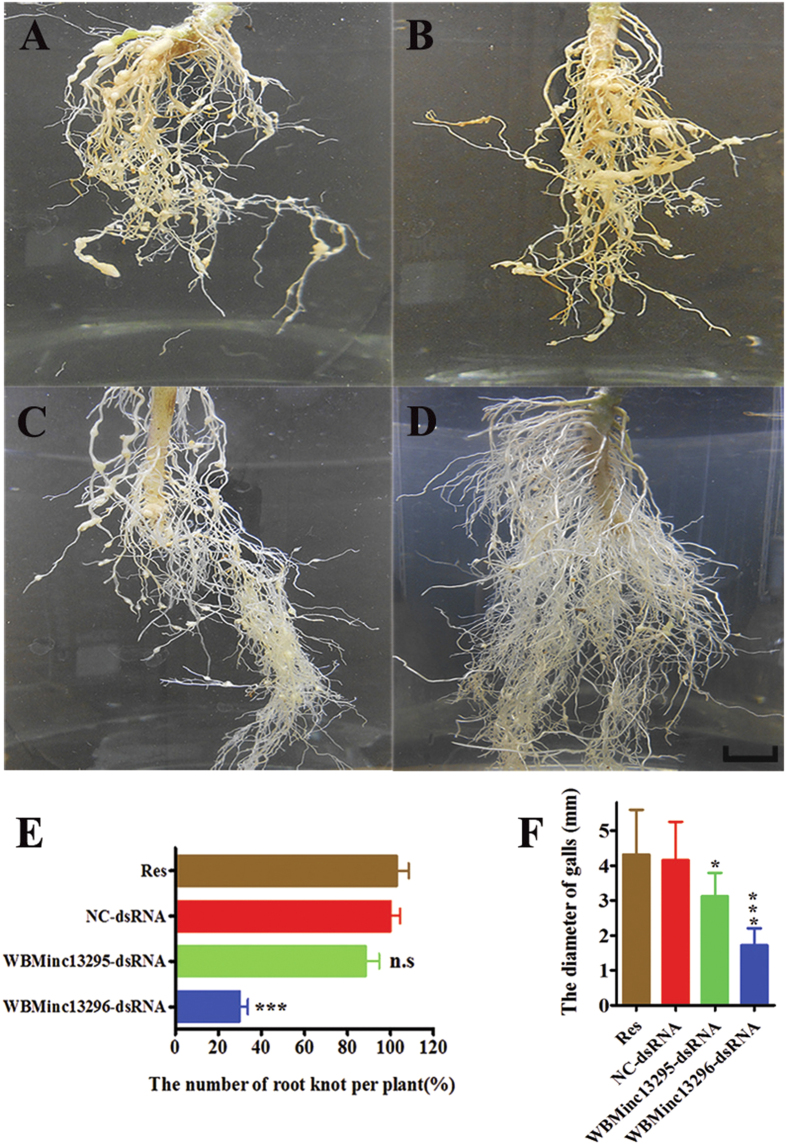
The effect of knockdown of *WBMinc13295* and *WBMinc13296* on pathogenicity of J2s in long period of time. **(A–D**) Representative symptoms of root knot on root system from different treatments (scale bar, 12 mm). (**A,B**) Refer to two controls, namely Res and NC-dsRNA, respectively. (**C,D**) Refer to WBMinc13295-dsRNA and WBMinc13296-dsRNA, respectively. (**E**) A decrease in the number of root knot was observed in root system after infection with the J2s with inactivation of *WBMinc13296*. (**F**) A decrease in the size of root knot was observed in root system after infection with the J2s with inactivation of *WBMinc13296* and *WBMinc13295*. Each bar value represents the mean ± SD of triplicate experiments. Res, resorcinol. Two-way ANOVA, ns: no significance; *P < 0.05; ***P < 0.001 versus control J2s (NC-dsRNA).

To study the role of *WBMinc13295* and *WBMinc13296* in the pathogenicity of *M. incognita in vivo*, nematode bioassays were performed. At 35 days post-infection (dpi), roots of tomatoes infected with J2s subjected to *WBMinc13296* dsRNA exhibited a significant decline in the number of galls and smaller size of galls compared to those infected with J2s subjected to NC-dsRNA. Meanwhile, the root system of tomatoes challenged with J2s treated with dsRNA of *WBMinc13295* exhibited a smaller size of galls, but a similar number of galls, compared to those incubated with J2s subjected to NC-dsRNA (Fig. 4A–F). Interestingly, similar results were observed in tests in which *M. incognita* J2s were subjected to siRNA of *WBMinc13296* or *WBMinc13295* (Figs S7 and S8). These results strongly suggested that inactivation of either *WBMinc13296* or *WBMinc13295* suppresses pathogenicity of J2.

### *WBMinc13296* and *WBMinc13295* are required for development and fecundity

**Figure 5:**
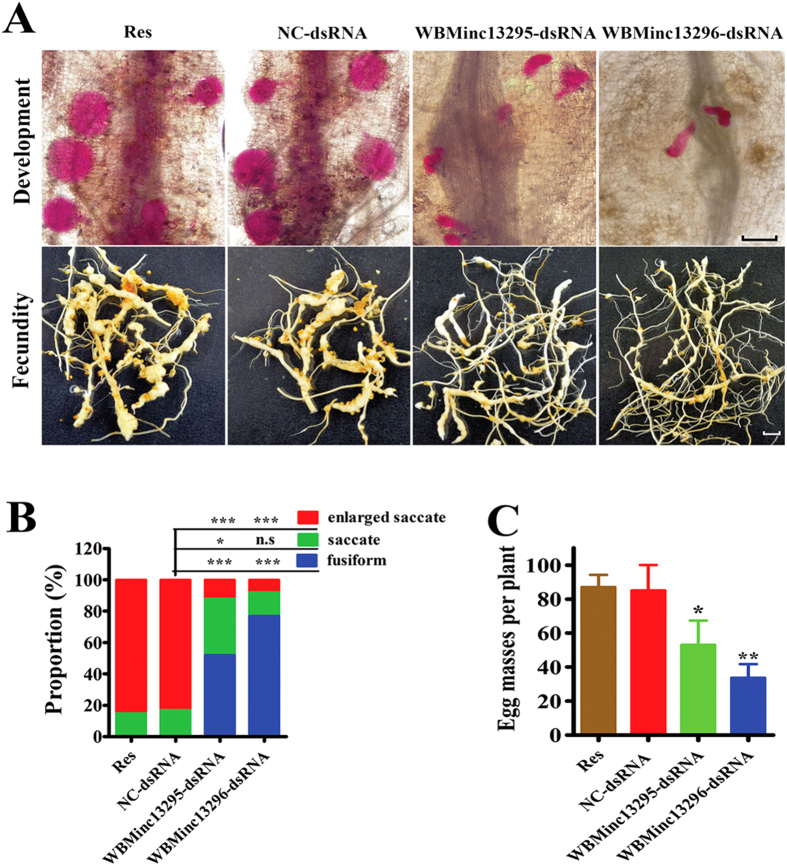
The effect of knockdown of *WBMinc13296* and *WBMinc13295* on development and fecundity in a long period of time. (**A**) Photographs displayed worms within different life-stage in tomato root (scale bar, 300 μm) and corresponding egg masses on tomato system (scale bar, 60 μm), respectively. The worms were stained with acid fuchsin and displayed red. (**B**) The proportion of treated nematode stages (fusiform, saccate and enlarged saccate). Worms with knock-down of *WBMinc13296* and *WBMinc13295* both displayed extremely increase and reduce in proportion of fusiform and enlarged saccate worms compare to controls, respectively. (**C**) The number of egg masses of worm per plant recovered after 45 dpi after treatment with dsRNA. Each bar value represents the mean ± SD of triplicate experiments (one-way ANOVA, n.s: no significance; *P < 0.05; **P < 0.01; ***P < 0.001).

The reduction in size of giant cells by knockdown of *WBMin13296* and *WBMinc13295* probably results from retarding development of *M. incognita*. Based on the association of shape and body-size of *M. incognita* with developmental stage, we classified worms within roots into three classes: fusiform (J2, J3, J4 and male), saccate females (young), and gravid females (mature) as described previously^36^,^37^. The relative growth rates and stages of development of worms in the population could be determined by such classification based on significant morphological differences. After 35 dpi, a reduction in the proportion of saccate females among worms treated by either *WBMinc13296* or *WBMinc13295* dsRNA was observed (Fig. 5A,B). Similar results were obtained from worms subjected to either *WBMinc13296* or *WBMinc13295* siRNA (Fig. S9).

The numbers of egg masses were significantly reduced in roots treated with J2 ingesting *WBMinc13296* and *WBMinc13295* dsRNA, compared with control treatment (NC-dsRNA) after 45 dpi (Fig. 5A,C). Similar results were observed in the testing of *WBMinc13296* and *WBMinc13295* siRNA (Fig. S9). Collectively, these data suggested that *WBMinc13296* and *WBMinc13295* are involved in the development and fecundity of *M. incognita*.

## Discussion

In this study, we demonstrated that the number of NR family members in plant parasitic nematodes is less than that in free-living and animal-parasitic nematodes^38^. The variation in the numbers of NRs appears to result from the niche specialization of internal plant parasites^39^. During the evolution of nematodes, soil-dwelling nematodes perhaps need a large number of NHRs with highly specialized functions to deal with ever-changing, multi-faceted conditions. In contrast, phyto-endoparasites inhabit a well-defined, homeostatic environment and the major challenges mainly come from the relatively constant defense of the host. The known nuclear receptors are split into six sub-families. The nuclear receptors with identified ligands are mainly diversified into three sub-families (I, II and III), whereas orphan receptors emerge in all sub-families, suggesting that nuclear receptors with identified ligands are probably derived from orphan receptors without ligands^40^. In *C. elegans*, DAF-12: NHR-8 might be derived from the orphan nuclear receptor NHR-48 during the second period of NR super-family gene duplication in which receptors of each group or sub-family generate paralogous versions potentially able to perform identical or similar functions^41^. Logically, this situation could be analogous to the situation in *M. incognita*. In *M. incognita*, NHR-48 has two copies without LBD: one is WBMinc13296 existing together with WBMinc13295 (LBD) at the chromosome level; the other one is the orphan WBMinc18589 emerging alone and showing a high level of identity with WBMinc13296 in nucleic and amino acid sequences (both > 97%). WBMinc18589 might still keep the ancestral trait of orphans like NHR-48 in *C. elegans*, whereas WBMinc13296 may have evolved towards DAF-12 and NHR-8 of *C. elegans* for its coexistence with WBMinc13295 (LBD) though they are not localized in the same gene. Collectively, the difference in NR1J+K between free-living nematodes and plant parasites may result from their difference in selection pressure and evolutionary rate. *M. incognita* is faced with relatively consistent selection pressure, leading to a slow evolutionary rate, whereas *C. elegans* confronts various selection pressures in the soil, resulting in a relatively high evolutionary rate. In other words, these results indicate that the expansion of the number of members of the NR1J+K group probably started during the evolutionary separation of *Caenorhabditis, Bursaphelenchus, Globodera,* and *Meloidogyne* and continued thereafter owing to various selection pressures^38^.

Another unique feature of the NR1J+K group NRs in plant parasites is that the two crucial domains (DBD and LDB) of their counterparts in *C. elegans* are always located in two different genes. *M. incognita* and *M. hapla* have two genes corresponding to encoding DBD and LBD of *nhr-48*, and *M. floridensis* and *G. pallida* have separate DBD and LDB of *daf-12* orthologs. The two genes containing the two crucial domains are reciprocal neighbors in *M. incognita* and perhaps in *M. hapla.* However, whether two counterparts are mutually proximate remains to be determined in *M. floridensis* and *G. pallida* due to the lack of high quality genomes. Based on the finding that the NR first emerged as an unit with recently recognizable DBD and LBD existing together in lower metazoans^42^, these separations seem to be intermediates in a hypothetical NRs evolutionary path in which the liganded NRs have been derived from orphan NRs by independently gaining ligand-binding capacity or LBD during subsequent evolution in models that are controversial to date^41^,^42^. There also appears to be compelling evidence of gene fusion co-evolution during the evolution of nematodes toward parasitism under certain selection pressures^39^,^43^, in which plant parasites have evolved at least three times independently within the nematodes^44^. In addition, the LBD-free *nhr-48* ortholog has two forms in *M. incognita*, namely *WBMinc13296* existing together with *WBMinc13295* and *WBMinc18589* emerging alone, which show high identities in nucleic and amino acid sequence with *WBMinc13296*. A reasonable explanation for this asymmetry is that the LBD-free orphan receptor *WBMinc18589* had been unable to gain LBD but *WBMinc13296* had in a sense during subsequent evolution of NRs^41^,^45^. Unlike *WBMinc13296,* knockdown of *WBMinc13295* seems not to influence motility and infection rate *in vitro*, suggesting that DBD can also function independent of LBD. A similar case was seen in CeDAF-12. CeDAF-12 can interact with its co-repressor DIN-1 to exhibit distinct functions in the absence of its ligand DA^18^. Clearly, the molecular mechanism underlying WBMinc13295 or WBMinc13296 mediated physiological functions needs to be investigated further in light of our current results.

Notably, divergence of ecological niches occupied by nematodes determines the type of ligands, which might account for the variance of LBD^46^. Like *C. elegans*, animal parasitic nematodes, such as *Ascaris suum, Ancylostoma* spp*, Necator* spp and *S. stercoralis*, are capable of selectively absorbing cholesterol from their animal host^47^,^48^. Thus, DA has an ability to suppress infectivity through reducing the iL3 population in *S. stercoralis* and inducing dauer recovery by activating the parasitic DAF-12s in a fashion similar to that observed with *C. elegans* DAF-12^20^,^21^. Plant obligate parasitic nematodes mainly rely on the plant host to get phytosterols, such as sitosterol, stigmasterol and ergosterol, which differ from cholesterol in the presence of methyl or ethyl groups at position 24 of the side chains^49^. These non-uniform feedings may account for the lower sequence similarity of LBD between plant-parasitic and free-living or animal-parasitic nematodes.

Based on the findings that, in addition to regulation of dauer formation in *P. pacificus, Ppa*-*daf-12* is also involved in the dimorphism of mouth forms and a mutation in *Ppa*-*daf-12* causes a significant decrease in the proportion of worms with eurystomatous mouth, which are essential for predation^28^. The reduction of infection by knockdown of *WBMinc13296* raises the intriguing possibility that LBD-free *nhr-48* of *M. incognita* might function in a similar fashion controlling the protrusion of the stylet. In addition, *nhr-48* of *C. elegans* is expressed in the pharyngeal gland of larvae and adults and also in the spermatheca beginning in L4^25^ and it has been reported that *nhr-48* is required to confine Y8A9A.2, a gland gene connected with feeding, to expression in g1P and g2 gland cells other than g1A gland cells. In the background of *nhr-48*(ok178), Y8A9A.2 expression was also observed in g1A gland cells, implying that *nhr-48* might play a role in the feeding of *C. elegans*^26^. A few studies have demonstrated that pharyngeal gland cell-expressed genes of plant parasites are required for initial interactions with the host^50^,^51^,^52^. This reduction in infectivity induced by the knock-down of LBD-free *nhr-48* in this study implied that LBD-free *nhr-48* appears to have a role in feeding similar to its role in *C. elegans*. This possibility is consistent with the previous observation that genes expressed in esophageal gland cells of *M. incognita* J2 are responsible for choosing and establishing a feeding site^9^. Collectively, in the absence of orthologs of DAF-12 and NHR-8, *M. incognita nhr-48* orthologs of *C. elegans* might share some common features and functions with *daf-12 and nhr-8* in *C. elegans*. Our work provides biological characterization of nuclear receptors in obligate plant parasites and thereby might provide targets for new resistant crops to prevent infection induced by plant parasites that cause numerous losses in world agriculture every year.

## Materials and Methods

### Collection and maintenance of nematodes

*M. incognita* isolates were maintained on cultures of susceptible tomato species (*Lycopersicon esculentum*) under greenhouse conditions with a temperature of approximately 25–30 °C. Roots were harvested from severely infected plants and washed rigorously in flowing tap water. To obtain freshly hatched J2s, eggs masses picked with dissecting needle were placed on a 50 μm nested sieve which was submerged in sodium hypochlorite (2.5% v/v) for a period of 2 min and then placed in DEPC-treated water in complete darkness at 25 °C. Then for the subsequent experiments, freshly hatched J2s were collected every two days for a period of eight days.

### Bioinformatic analysis

The published *M. incognita, M. hapla, B. xylophilus, C. elegans, P. redivivus* and *P. pacificus* genomes, and the predicted transcriptomes and proteomes used in this study, were downloaded from ftp://ftp.wormbase.org/pub/wormbase/species/. Counterparts of *G. pallida* and *M. floridensis* were obtained from http://www.sanger.ac.uk/sequencing/Globodera/pallida/ and http://nematodes.org/genomes/meloidogyne_floridensis/, respectively. The sequences of *C. elegans* DAF-12, NHR-8 and NHR-48 were obtained from RNAi Date Base (RNAiDB) (http://www.rnai.org/) and local BLASTp searches were separately carried out against the proteomes. The high score return sequences in each hit were further confirmed through performing local BLASTp reciprocally against the *C. elegans* proteome. In addition, the homologues were carried out in Inter-ProScan (http://www.ebi.ac.uk/Tools/InterProScan/), NCBI Conserved Domain Database (CDD) software (http://www.ncbi.nlm.nih.gov/Structure/cdd/cdd.shtml) and smart (http://smart.embl-heidelberg.de/) to detect conserved domains. To further identify candidates, multiple sequence alignments of homologs were assembled using ClustalW with default parameters^53^. The conserved DBD and variable LBD within the homologues were aligned, respectively. The phylogeny trees of DBD and LBD were constructed using Mega6.0^31^, respectively. The parameter values were determined by strength and the bootstrap replications was 1000. In addition, phylogeny trees were also constructed with maximum parsimony^53^.

### Synthesis of dsRNA *in vitro*

Total RNA was extracted according to manufacturer’s instructions of RNA-prep pure Micro Kit and quantified with a spectrophotometer (ND-2000, NanoDrop). Then, 2 μg total RNAs extracted from J2s and females were used as matrices for reverse transcription with PrimeScript 1st Strand cDNA Synthesis Kit (TaKaRa). Pairs of T7-labeled gene-specific primers (Table S3) were used to amplify conserved fragments of homologues. For all PCR reactions, standard conditions were applied for a maximum of 33 cycles and annealing temperatures were determined for individual pairs of primers. PCR products were separated on 2% agarose gel by electrophoresis and stained in ethidium bromide. Then the amplified cDNA fragment was subcloned into pMD18-T Simple Vector (TaKaRa Biotech) and both strands were sequenced by BGI. For synthesis of dsRNA of target genes, amplified cDNA fragments were purified with the guanidinium phenol extraction method and concentrated to 2 μg/μl quantified with a spectrophotometer (ND-2000, NanoDrop). We obtained 526 bp of *WBMin13296* and 488 bp of *WBMinc13295* fragments from *M. incognita*, respectively. Using the fragments from *WBMin13296* and *WBMin13295* as templates, their dsRNAs were synthesized in a single reaction using MEGA script RNAi kit (Ambion, USA) according to the manufacturer’s instructions. A sequence of approximately 500 bp with no similarity in the *M. incognita* genome^3^ as confirmed by BLASTn searches was employed as a negative control (Table S2). All dsRNAs were stored at −80 °C in 50 μg aliquots until use.

### Synthesis of siRNA

On the basis of the siRNA design guideline as previously described^54^,^55^,^56^, from plentiful siRNA candidates, four and three discrete siRNAs with the top comprehensive scores were selected as RNAi triggers against *WBMin13296* and *WBMin13295*, respectively (Table S1). These selected siRNA with 30–50% GC content started with AA followed by 19 nt and no more than 14 nt contiguous base pair identity with other coding sequences. A randomized siRNA duplex with no similarity within the *M. incognita* genome was employed as a control. The above siRNAs were synthesized by GenePharma Co., Ltd (Shanghai China).

### Examination of the effect of J2 uptake *in vitro*

For soaking conditions, two methods were employed in the present study. The first one was performed as described by Huang *et al*.^9^. The second was a two-stage stimulated ingestion approach slightly modified from the first. Briefly, freshly hatched J2s of *M. incognita* were incubated in 0.25 × M9 buffer containing 1% resorcinol, 0.1 mg/ml FITC isomer I, 0.05% gelatin, and 3 mM spermidine for 2 h in the dark at room temperature on a rotator. Afterwards, incubated J2s were thoroughly washed at least three times with 0.25 × M9 buffer to remove residual resorcinol and then soaked in 0.25 × M9 buffer containing 0.1 mg/ml FITC isomer I, 0.05% gelatin, and 3 mM spermidine for 24 h under complete darkness on a rotator. Then, about 200 FITC-labeled J2s randomly picked from the two methods were observed with a fluorescence microscope (Olympus, Melville, NY) to monitor the vitality of J2 and the intensity of the FITC uptake. Images were captured using an Axiocam camera equipped with a filter (emission 488–525 nm). All images were captured using identical settings and exposure times. Image J software was used to quantify the fluorescence intensity of FITC. Equal regions of the nematodes’ bodies were selected, and the intensity of staining of granules within the selected regions was measured three times per nematode. More than ten worms were observed to calculate the mean fluorescence intensity and the data were analyzed with Bonferroni post-tests using GraphPad PRISM Version5 package for Windows (GraphPad Software, Inc).

The second soaking method was employed for ingestion of siRNA or dsRNA in subsequent experiments. Control samples of J2s were incubated in the same solution with non-native siRNA (non-native dsRNA) or without resorcinol or dsRNA (siRNA) and each treatment was carried out in triplicate. After soaking, treated J2s were washed five times by centrifugation at 8000 rpm for 2 min and suspended in 100 μl of DEPC-treated water for about 24 h at room temperature to allow for subsequent experiments.

### Quantitative PCR (qPCR) analysis

Real-time quantitative RT-PCR was carried out in the Roche LightCycler 480 using SuperReal Pre-Mix Plus (SYBR Green) Reagents (Applied Bio-systems). The amplifications were performed using 1:25 diluted first-strand cDNA from each treatment with final primer (Table S3) concentrations of 0.5 μM each in individual 20 μl reaction volume for qPCR analysis of target transcripts. *M. incognita* actin gene (GenBank accession no. BE225475) was employed as a reference gene for relative quantification of target transcript expression. The data of Ct were analyzed by one-way ANOVA and Bonferroni tests using Graph-Pad Prism 5.

### Attraction assays and penetration of J2 in *Pluronic* gel medium

A volume of 60 μl of 23% Pluronic gel at 16 °C with 40 J2 from individual treatments was pipetted onto glass plate (100 × 75 mm) as Wang *et al*. described^34^. A sterile 5-day-old tomato root tip 1.5 cm in length with few root hairs was placed in the centre of the gel and covered with a glass slide. To prevent the dehydration of the root, wet filter paper was lined with glass slides and the glass plate was transferred into 200 mm plate with 10 ml water at room temperature. Every two hours after the start of the assay, individual J2 near the root tip (the terminal 1.5 mm of the root tip surface) were monitored and photographed. In addition, individual infected roots were stained to dissect J2 at 12 h and 48 h as previously described^35^,^57^. Nine to eleven replicates were employed for each treatment and each treatment was repeated three times. In this experiment, photos were taken with an Olympus BX50 microscope. Attraction rate and invasion rate of worms treated with NC-dsRNA or NC-siRNA in 6 h and 48 h were standardized to 100%, respectively and the data were analyzed as previously described.

### Migration assay

Approximately 1000 J2s treated with dsRNA or siRNA were applied in triplicate into glass columns (5 mm internal diameter) sealed at one end with nylon net (300 mesh) and containing 50 mm moistened sand (grain diameter 0.25–1.0 mm) as described previously^13^. The glass tube containing sand was placed vertically in a 24 Well Cell Culture Cluster (Lifesciences, USA) with 1 ml water added to the tube and sand column. In following migration experiments, the number of nematodes finishing migration of the sand column was counted every four hours for a total 48 h. Subsequently, the nematodes unable to complete the migration in all treatments were washed out and counted. Migration percentages of nematode soaking treated with negative control dsRNA or siRNA were standardized to 100%. The results of migration assays were analyzed by one-way ANOVA and Bonferroni post-tests using GraphPad PRISM Version 5 package for Windows (GraphPad Software, Inc.). Data with probabilities of less than 5% (P < 0.05) were deemed statistically significant.

### Infection with host

Ahead of incubation with nematodes, 15-day old tomato plants grown in sand were watered with about 30 ml of spring water. After watering, approximately 800 J2 of *M. incognita* from each treatment were applied to the sand covering the root network in 1 ml DEPC treated water (n = 5 plants per treatment). Plants were sequentially grown under greenhouse conditions with a cycle of 16 h /8 h light/dark for 35 and 45 days, when their infection and reproductivity were analyzed, respectively. Tomato plants with their growth medium were removed from pots and then gently rinsed in fresh water to remove sand from roots. After the root-knots and egg masses were counted, the diameter of gall was determined by vernier caliper, and then, the roots were stained with acid fuchsin as previously described. Root sections from each plant of each treatment were put on glass microscope slides and viewed with an Olympus SZ X16 microscope. Fusiform, saccate and enlarged saccate worms within root segments were counted per root system. Infection rate of nematode soaking treated with negative control dsRNA or siRNA were standardized to 100% and then data were analyzed by one-way ANOVA and Tukey’s Honestly Significant Difference (HSD) post-test using GraphPad PRISM Version 5 package for Windows (GraphPad Software,Inc.). Data with probabilities of less than 5% (P < 0.05) were deemed statistically significant.

## Additional Information

**How to cite this article**: Lu, C.-J. *et al*. Nuclear receptor *nhr-48* is required for pathogenicity of the second stage (J2) of the plant parasite *Meloidogyne incognita. Sci. Rep.* **6**, 34959; doi: 10.1038/srep34959 (2016).

## Supplementary Material

Supplementary Information
