# Supplementary material for: Nuclear receptor nhr-48 is required for pathogenicity of the second stage (J2) of the plant parasite Meloidogyne incognita
Source: Sci Rep. 2016 Oct 20;6:34959. doi: 10.1038/srep34959 (PMC5071846; doi:10.1038/srep34959)

**Nuclear receptor *nhr-48* is required for pathogenicity of the second stage (J2) of the plant parasite *Meloidogyne incognita***

Chao-Jun Lu^1^, Bao-Yu Tian^2^, Yi Cao^3^, Cheng-Gang Zou^1,*^, Ke-Qin Zhang^1,*^

^1^ State Key Laboratory for Conservation and Utilization of Bio-Resources in Yunnan, Yunnan University, Kunming, Yunnan 650091, China

^2^ College of Life Science, Fujian Normal University, Fuzhou, Fujian 350108, China

^3^ Key Laboratory of Molecular Genetics, Guizhou Academy of Tobacco Science, Guiyang, Guizhou 550081, China

^*^Correspondence and requests for materials should be addressed to K-Q. Z. (email: kqzhang1@ynu.edu.cn), or C-G. Z. (email: chgzou@ynu.edu.cn).

**Supplementary Tables**

**Table S1. siRNAs** used in this study.

| Target gene | target site | sense（5'-3'） | antisense（5'-3'） |
| --- | --- | --- | --- |
| *WBMinc13296* | 186 | GCAACUUUCUGAUCGUUAUTT | AUAACGAUCAGAAAGUUGCTT |
|  | 268 | GCUGUGAGUUCUUCUGUAUTT | AUACAGAAGAACUCACAGCTT |
|  | 414 | GGAAUUUGAUAGAGGUCAUTT | AUGACCUCUAUCAAAUUCCTT |
|  | 1029 | GGAGAUGGAUCAACAAGAATT | UUCUUGUUGAUCCAUCUCCTT |
| *WBMinc13295* | 546 | CCAACAACCUCCAAAUAUUTT | AAUAUUUGGAGGUUGUUGGTT |
|  | 1423 | GCUGAAUUGUCAAAGGCUATT | UAGCCUUUGACAAUUCAGCTT |
|  | 1767 | GCGUCGAAAUGAAACAAUUTT | AAUUGUUUCAUUUCGACGCTT |
| Control | NC-siRNA | UUCUCCGAACGUGUCACGUTT | ACGUGACACGUUCGGAGAATT |

**Table S2.Template sequence of negative dsRNA in the study.**

5'-CGAATTCTAATACGACTCACTATAGGGGTATAATAGTGGCAGCAGTGAAATGCCTGCGGCTCAAACCATCAAGCAGGAGTACCACAATGGCTATGGTCAGCCGACACATCCTGGATACGGATTTAGCGCCTAATGCCAACAGAATCCGATAGCCCATCCCGGCCAGAATCCACACCAGACACTGCAGAATTTCTTTAGCCGCTTCAATGCCGTCGGTGATGCGAGTGCGGGAAATGGTGGAGCGGCTTCCATCTCAGCCAACGGATCGGGTTCGTCTTGCAACTACAGTCATGCGAATCATCATCCGGCGGAGCTGGACAAGCCGTTGGGCATGAATATGACACCGTCGCCCATCTACACCACCGACTACGATGACGAGAACAGCAGTCTCAGCTCCGAGGAGCACGTCCATGCGCCCCTCGTCTGCTCCTCCGCCCAATCCTCCAGACCATGCCTCACTGGGCCTGCAAGGCGTGCAAAAAGAAGCCCTATAGTGAGTCCGTATTAGAATTCG-3'

**Table S3. Primers used in this study.**

| **Primers name** | **sequence(5’-3’)** |
| --- | --- |
| WBMinc13296F | *GGATCC*TAATACGACTCACTATAGGGAGAGAGGAAGATGAAGAGGAGGAA |
| WBMinc13296R | *CTCGAG*TAATACGACTCACTATAGGGAGCAAGAGCGGCATTTACAGAA |
| WBMinc13295F | *GGATCC*TAATACGACTCACTATAGGGAGCACAAGCGGTGCTTCGTTTT |
| WBMinc13295R | *CTCGAG*TAATACGACTCACTATAGGGAGATCGCACTCTGCAAATCAGC |
| WBMinc13296RT-F | CAAGTGCTTCTTGTGCCTCT |
| WBMinc13296RT-R | TTCGTTGCTGGACGTTTC |
| WBMinc13295RT-F | AAGACTTCGGCAACAACT |
| WBMinc13295RT-R | TTAATCGACGCAAATCAG |
| beta-actin RT-F | CGCAATCTTGCGTCTTGACT |
| beta-actin RT-R | CACGGACATCTCACGTTCGG |
| WBMinc13296/186F-siRNA | CCCAGATAGTGGTGCAGATGG |
| WBMinc13296/186R-siRNA | GCTGTATTCTTCACTTGCCCA |
| WBMinc13296/268F-siRNA | CTAATTCAATGCCGCCTGTTG |
| WBMinc13296/268R-siRNA | ACCTCTATCAAATTCCTCATCATCC |
| WBMinc13296/414F-siRNA | CTAATTCAATGCCGCCTGTTG |
| WBMinc13296/414R-siRNA | ACCTCTATCAAATTCCTCATCATCC |
| WBMinc13296/1024F-siRNA | AATGGAGAACGTCGTGCGGC |
| WBMinc13296/1024R-siRNA | CTCGTTTGTTGTTGAGTTAAAGCTA |
| WBMinc13295/546F-siRNA | TTAACACCTGGGAGTACA |
| WBMinc13295/546R-siRNA | TTAGTTGAAGGAGCTGGA |
| WBMinc13295/1423F-siRNA | TTGTCAAAGGCTAACGAA |
| WBMinc13295/1423R-siRNA | TTAATCGACGCAAATCAG |
| WBMinc13295/1767F-siRNA | TTTACTGTTGCTATTCTACG |
| WBMinc13295/1767R-siRNA | GTAACAGAAATCGCACTC |

Underlined letters refer to T7 promoter sequences

In the case of detection of *WBMinc13296* expression after RNAi, 268 and 414 use the same primer

**Supplementary Figures**

**Figure S1. The structures and alignments of *nhr-48* orthologs and the production of corresponding dsRNA.** A: *WBMinc13295* and *WBMinc13296* locate in the same contig-MiV1ctg662 and separated by a segment with 893 bp nucleotide in length, whereas, *WBMinc18589* locate in another contig-MiV1ctg2093. As for *M. hapla nhr-48* orthologs of *C. elegans* locate in two successive contigs, namely *MhA1_Contig1158* and *MhA1_Contig1159*, even segregated by genomic fragment appearing to come from *Streptomyces spp*. B and C: Multiple alignment of the identified and deduced amino acid sequences of NR1J+K DBD and LBD from free-living and plant parasitic nematodes. The # indicates Cys residues consisting zinc finger of DBD, which is composed of two zinc finger containing four Cys. Black box indicates base-contact residues (ESCKAFFR) unique among NR1J+K NHRs group. Amino acid residues labelled with asterisk are conserved and importance of constituting LBD in NR1J+K. D: Determination of *WBMin13295* and *WBMinc13296* gene expression through amplification from J2 and female. M: marker, AF: adult female, J2: second stage of juveniles. E: Amplification of dsRNAs using conserved cDNA of *WBMin13296* and *WBMinc13295* as completes extracted from J2, M: marker, line 1: the dsRNA of *WBMinc13296*, line 2: the dsRNA of *WBMinc13295* and line 3: negative control dsRNA.


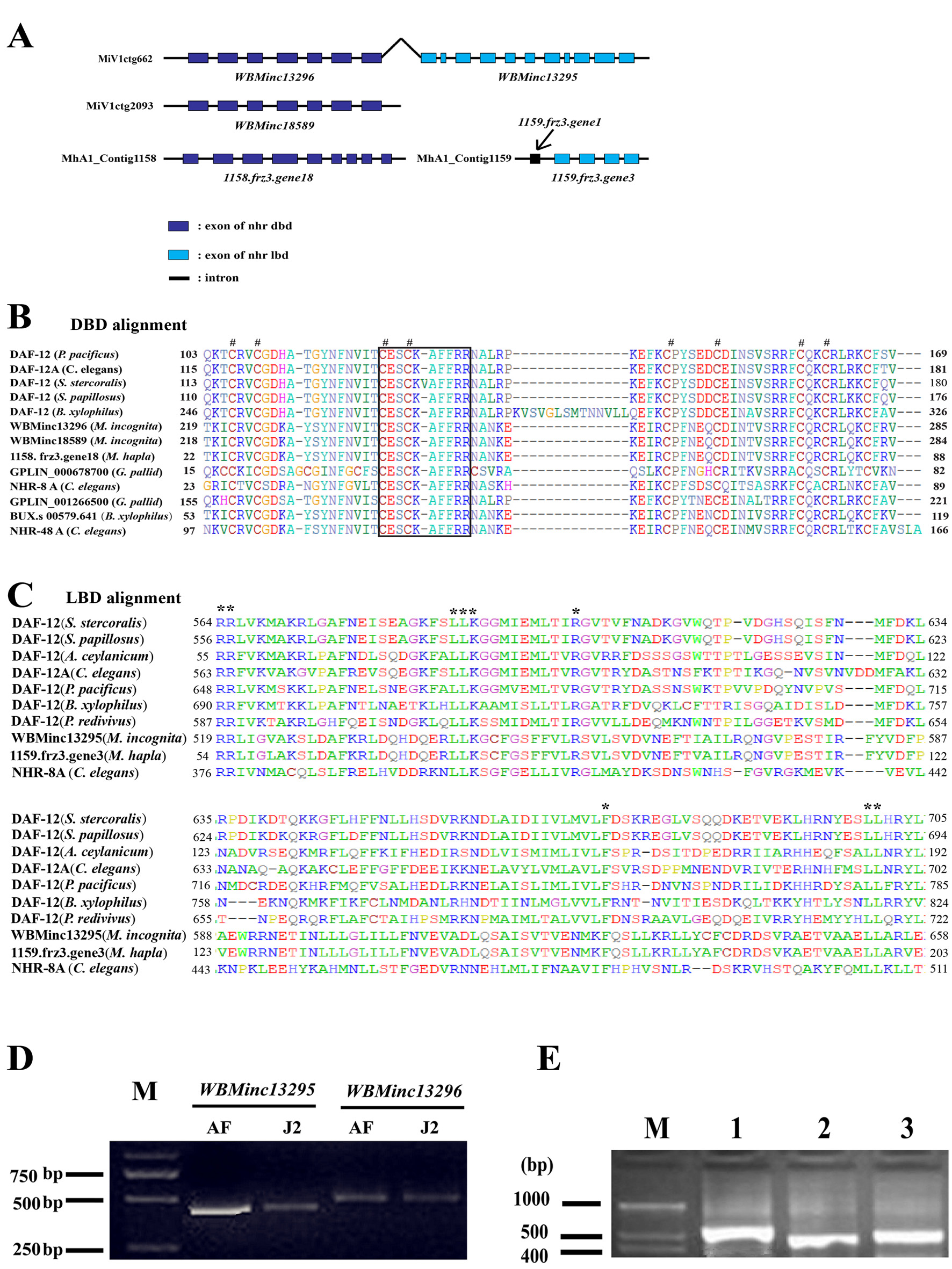


**Figure S2. Comparison of oral take quantity and mortality of J2s treated with two different approaches.** A: Fluorescence microscopy showing ingestion of FITC in the treated J2s (Scale bar, 50 μm) and mortality of the treated J2s (Scale bar, 200 μm). B: statistical analysis of fluorescence intensity of FITC in the treated J2s (Student’s t test; *, *P* < 0.05). C: statistical analysis of survival rate of treated J2s (Student’s t test; **, *P* < 0.01).


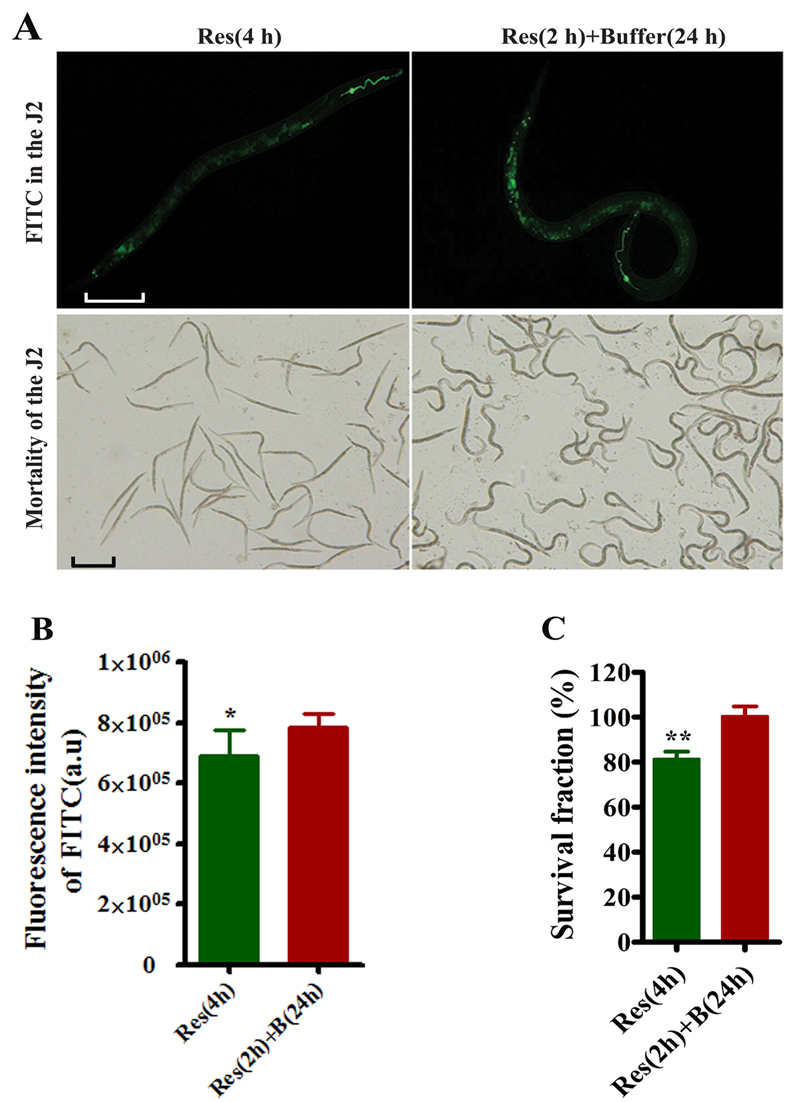


**Figure S3. Relative transcript abundance of *WBMinc13295* and *WBMinc13296* after RNAi silencing of using dsRNA or siRNA in pre-parasitic *M. incognita* J2.** A: NC-dsRNA as control, WBMinc13295-dsRNA and WBMinc13296-dsRNA served as treatments. B: Relative transcript abundance of *WBMinc13295*, NC-siRNA as control and the test of three siRNAs worked as treatments. C: Relative transcript abundance of *WBMinc13296*, NC-siRNA as control and the test of four siRNA acted as treatments. Each bar value represents the mean ± SD of triplicate experiments (one-way ANOVA,***, *P*<0.001).


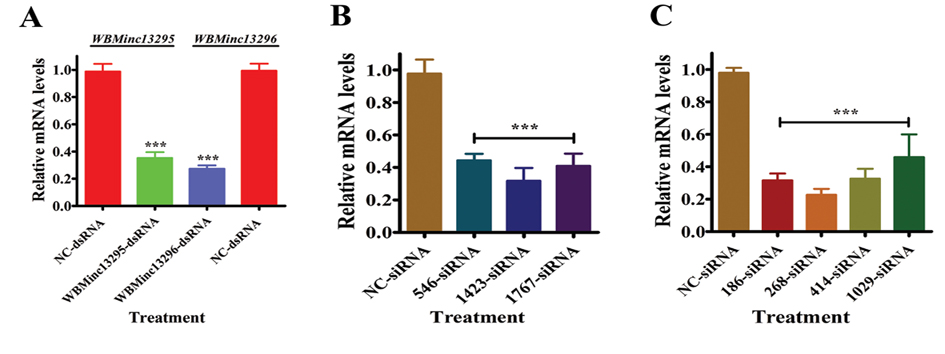


**Figure S4. Attraction of the J2s treated with siRNA of *WBMinc13295* and** ***WBMinc13296* in Pluronic gel, respectively.** A and B: photographs displayed attraction of the treated J2s siRNA of *WBMinc13295* and *WBMinc13296* response to tomato root tip at period of 2 h and 6 h (Scale bar, 400 μm). C and D: J2s treated with siRNAs *WBMinc13295* and *WBMinc13296* did not exhibited obvious difference in response to host root tip compared to control J2s. Each bar value represents the mean ± SD of triplicate experiments (Two-way ANOVA, n.s: no significance).


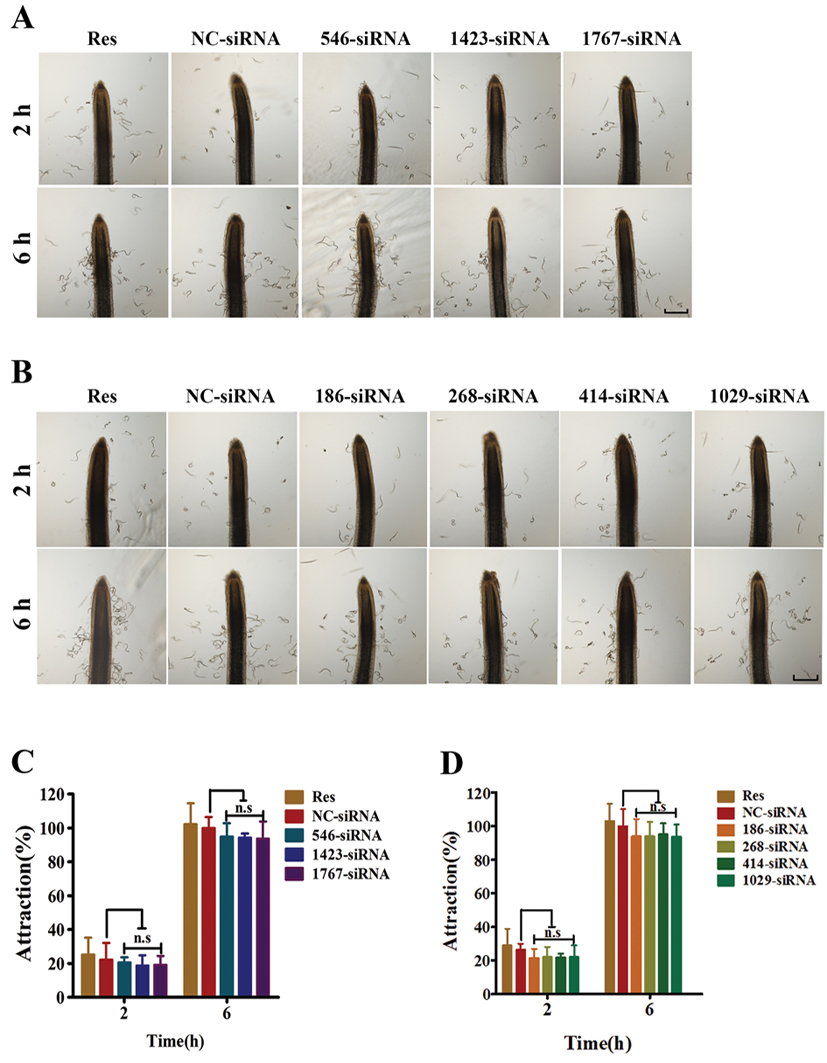


**Figure S5. The invasion of the J2s treated with siRNA of *WBMinc13295* and *WBMinc13296* in Pluronic gel.** A: photographs displayed J2s treated with siRNA of *WBMinc13295* in tomato root at of period of 12 h and 48 h (scale bar, 300 μm). B: photographs displayed J2s treated with siRNA of *WBMinc13296* in tomato root at of period of 12 h and 48 h (scale bar, 300 μm). C: J2s with knock-down of *WBMinc13295* did not exhibit obvious decrease in invasion rate compared to control at of period of 12 h and 48 h. D: J2s with knock-down of *WBMinc13296* displayed obvious decrease in invasion rate compared to control at of period of 12 h and 48 h. Each bar value represents the mean ± SD of triplicate experiments (Two-way ANOVA, **, *P*<0.01; ***, *P*<0.001).


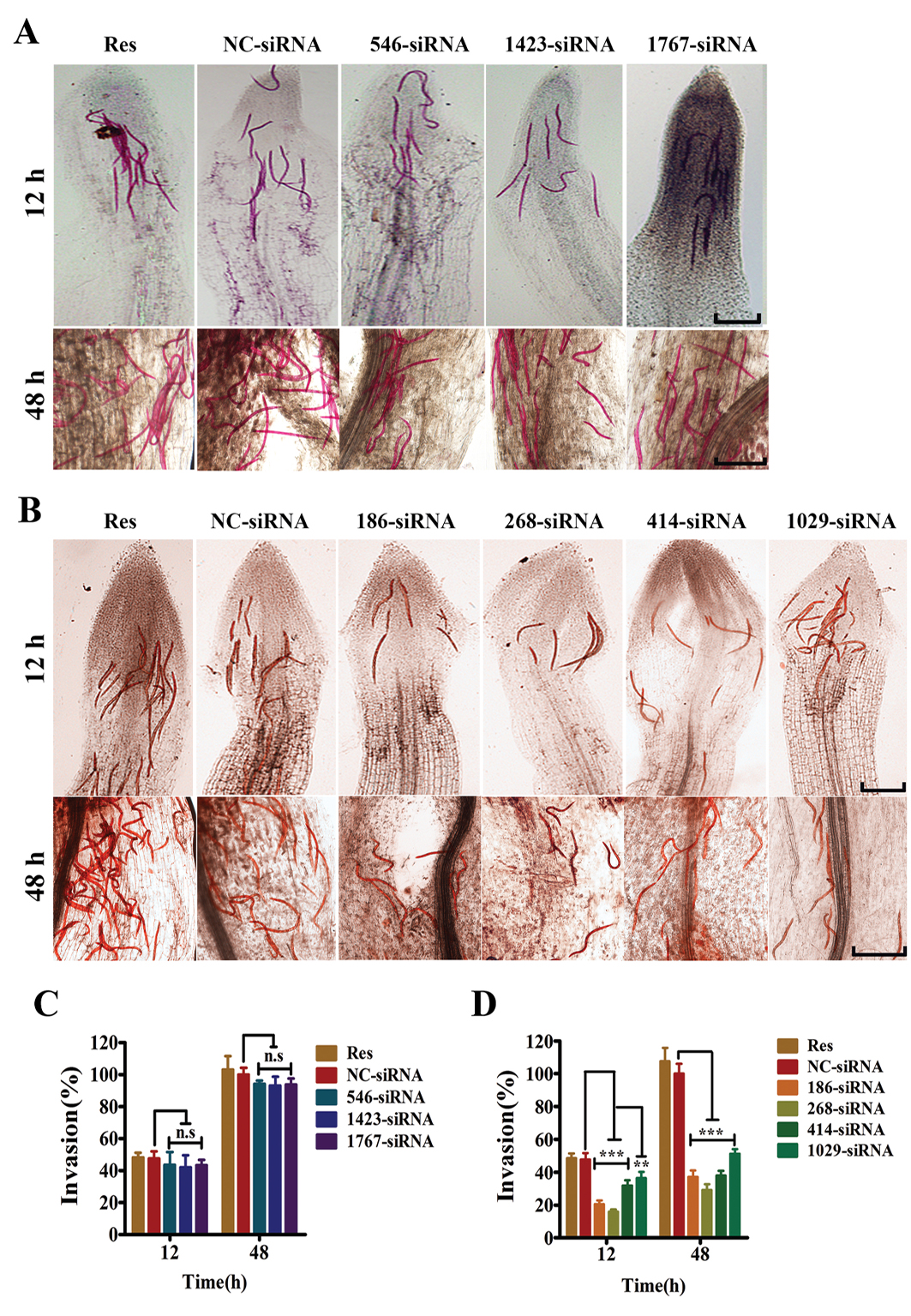


**Figure S6. Migration rate of J2s treated with dsRNA or siRNA** **of** ***WBMinc13295* and *WBMinc13296* in sand column.** A: J2s treated with dsRNA of *WBMinc13296* has begun to show significantly enhanced migration rate at 12 h after assay starting compared to two controls (Res and NC-dsRNA). B: J2s treated with three discrete siRNAs of *WBMinc13295* show comparable migration rate compare to that of two controls in entire migration time-course assay. C: J2s treated with four discrete siRNAs of *WBMinc13296* have begun to show significantly enhanced migration rate at 16 h after assay starting compared to two controls­­­. Each bar value represents the mean ± SD of triplicate experiments (Two-way ANOVA, **, *P*<0.01; ***, *P*<0.001).


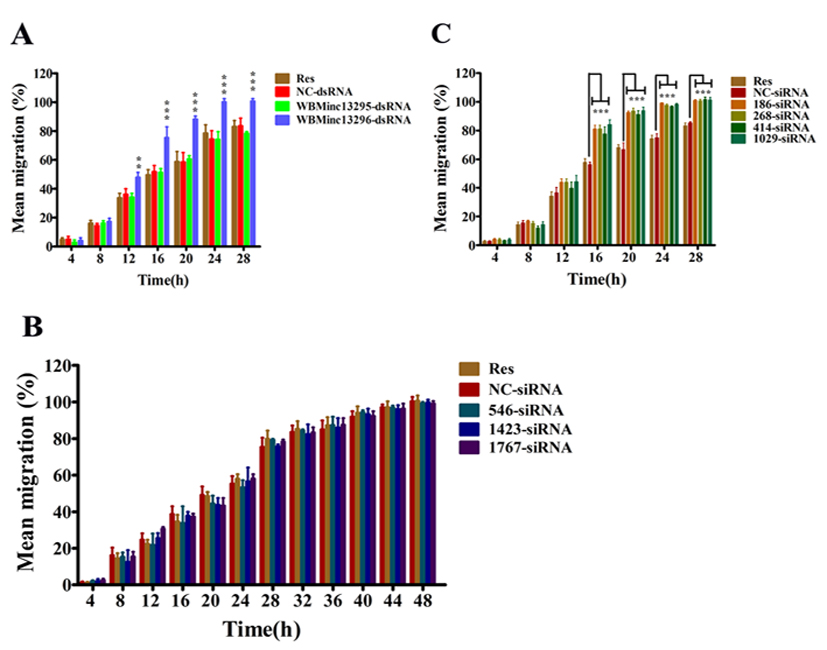


**Figure S7**.The effect of knockdown of *WBMinc13295* on pathogenicity of J2s in long period of time. A-E: Representative symptoms of root knot on root system from different treatments (scale bar, 12 mm). A and B refer to two controls, namely Res and NC-siRNA, respectively. C, D and E represent the 546-siRNA, 1423-siRNA and 1767-siRNA of *WBMinc13295*, respectively*.* F: The J2s with inactivation of *WBMinc13295* did not display significant decrease in the number of root knot compared to control J2s. G: The J2s with inactivation of *WBMinc13295* exhibited significant decrease in the size of root knot compared to control J2s. Each bar value represents the mean ± SD of triplicate experiments (Two-way ANOVA, n.s: no significance, *, *P*<0.05; **, *P*<0.01; ***, *P*<0.001).


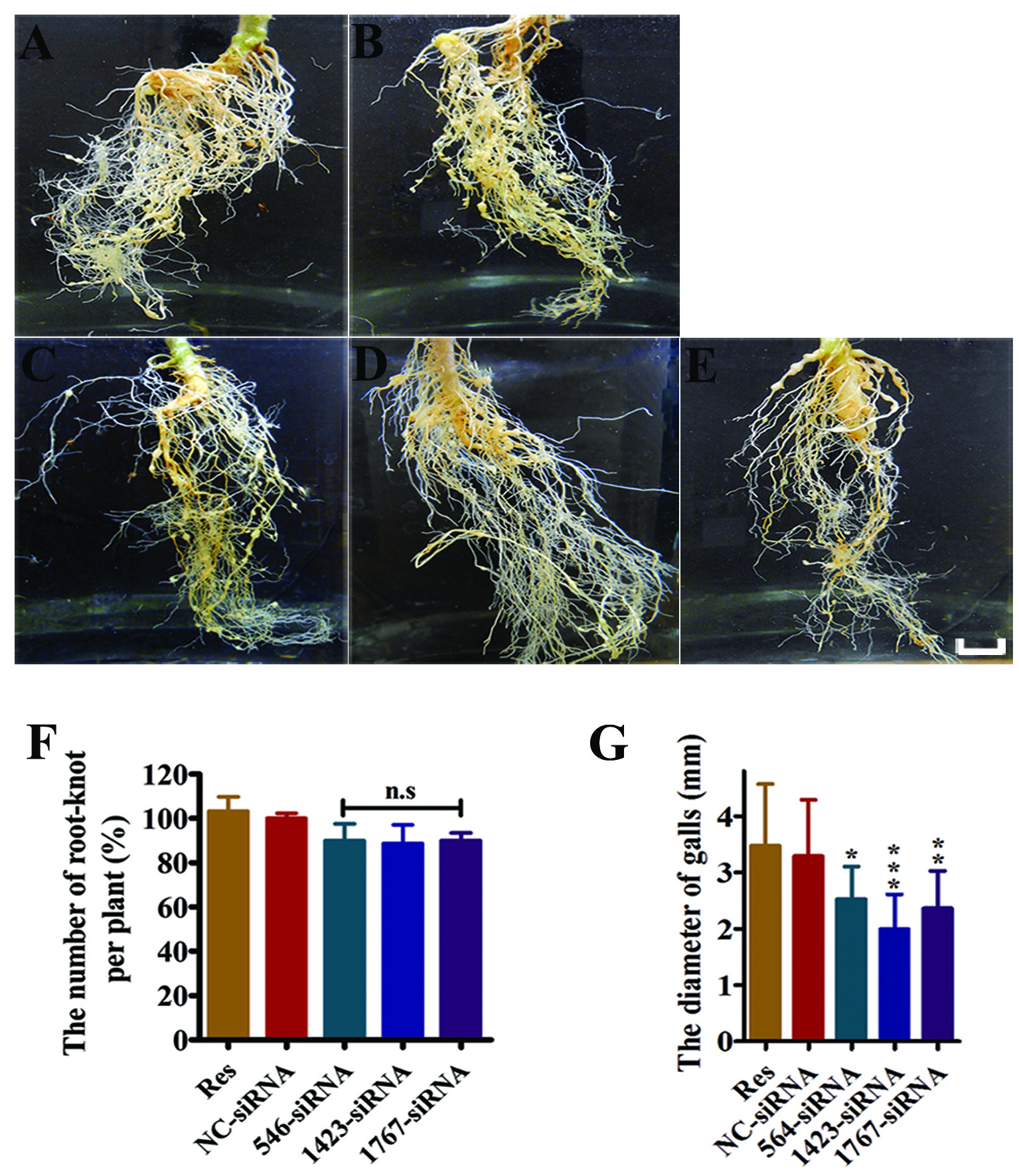


**Figure S8. The effect of knockdown of *WBMinc13296* on pathogenicity of J2s in long period of time.** A-F: Representative symptoms of root knot on root system from different treatments (scale bar, 12 mm). A and B refer to two controls, namely Res and NC-siRNA, respectively. C, D, E and F represent the 186-siRNA, 268-siRNA 414-siRNA and 1029-siRNA of *WBMinc13296*, respectively. G: The J2s with inactivation of *WBMinc13296* shown significant decrease in pathogenicity compared to control J2s. H: The J2s with inactivation of *WBMinc13296* displayed significant decrease in the size of root knot compared to control J2s. Each bar value represents the mean ± SD of triplicate experiments (Two-way ANOVA, *, *P*<0.05; **, *P*<0.01; ***, *P*<0.001)


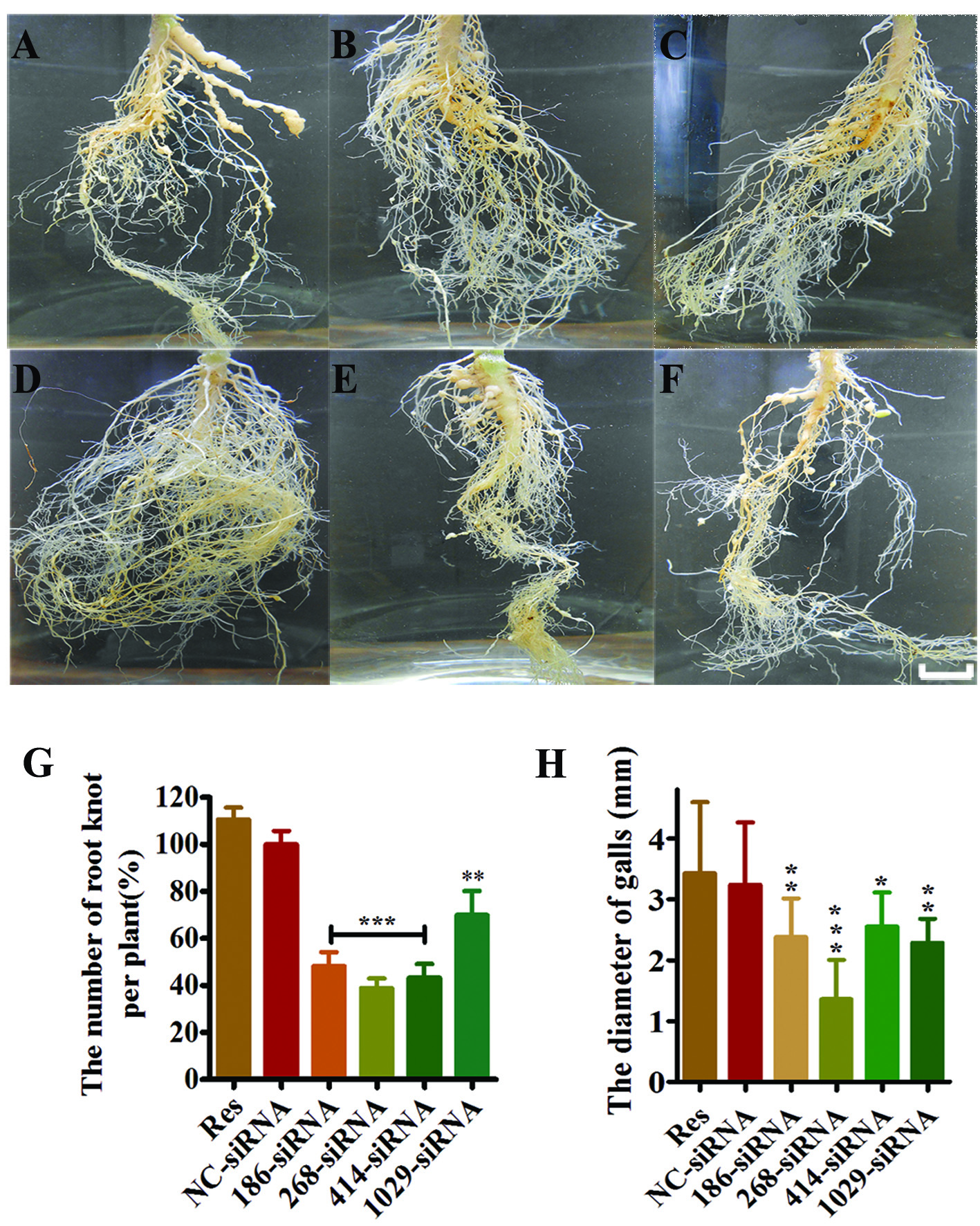


**Figure S9. The inactivation of *WBMinc13295* and *WBMinc13296* had influenced on development and fecundity in a long period of time.** A: photographs displayed *WBMinc13295* siRNA treated worms within different life-stage in tomato root (scale bar, 300μm) and corresponding egg masses on tomato system (scale bar, 60 μm), respectively. B: photographs displayed *WBMinc13296* siRNA treated worms within different life-stage in tomato root (scale bar, 300 μm) and corresponding egg masses on tomato system (scale bar, 60 μm), respectively. C: The proportion of *WBMinc13295* siRNA treated nematode stages (fusiform, saccate and enlarged saccate). Worms with knock-down of *WBMinc13295* displayed extremely increase and reduce in proportion of fusiform and enlarged saccate worms compare to controls. D: The number of egg masses of worm per plant recovered after 45 dpi after treatment with siRNA of *WBMinc13295*. E: The proportion of *WBMinc13296* siRNA treated nematode stages (fusiform, saccate and enlarged saccate). Worms with knock-down of *WBMinc13296* displayed significant increase and reduce in proportion of fusiform and enlarged saccate worms compare to controls. F: The number of egg masses of worm per plant recovered after 45 dpi after treatment with siRNA of *WBMinc13296*. Each bar value represents the mean ± SD of triplicate experiments (one-way ANOVA, n.s: no significance; *, *P*<0.05; **, *P*<0.01; ***, *P*<0.001).


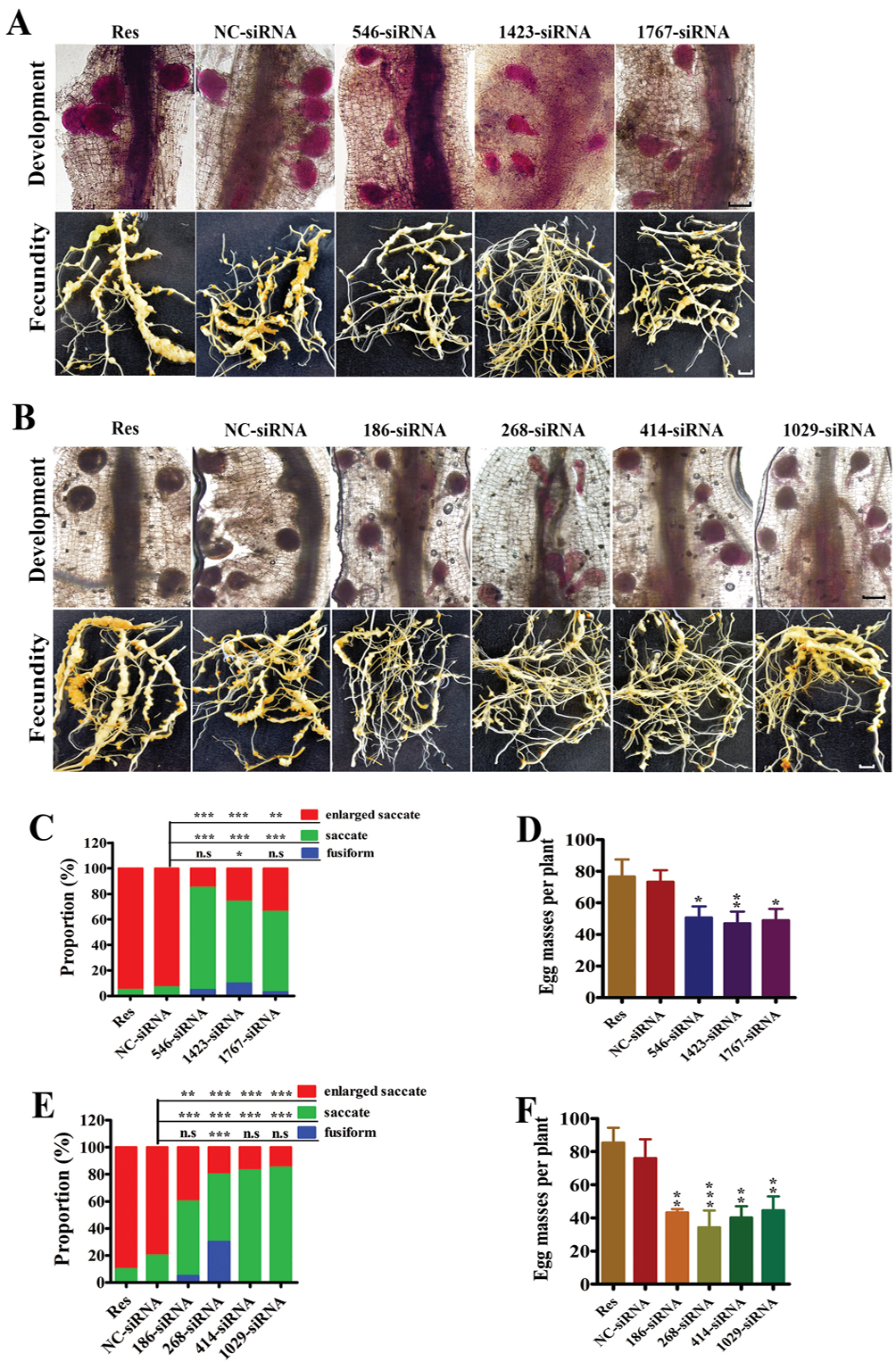

Supplement: Supplementary Information [file srep34959-s1.doc]
